# Predictive and Reactive Control During Interception

**DOI:** 10.3390/brainsci16030322

**Published:** 2026-03-18

**Authors:** Mario Treviño, Nathaly Martín, Andrea Barrera, Inmaculada Márquez

**Affiliations:** 1 Laboratorio de Plasticidad Cortical y Aprendizaje Perceptual, Instituto de Neurociencias, Universidad de Guadalajara, Colonia Arcos Vallarta, Guadalajara 44130, Mexico; maria.marquez@academicos.udg.mx; 2 Departamento de Ciencias Médicas y de la Vida, Centro Universitario de la Ciénega, Universidad de Guadalajara, Ocotlán 47810, Mexico; 3 Departamento de Psicología, Centro Universitario de la Ciénega, Universidad de Guadalajara, Ocotlán 47810, Mexico; 4 Laboratorio de Neurofisiología, Departamento de Bioingeniería Traslacional, Universitario de Ciencias Exactas e Ingenierías, Colonia Olímpica, Guadalajara 44430, Mexico

**Keywords:** visuomotor interception, predictive processing, reactive control, gaze–hand coordination, visual occlusion

## Abstract

**Highlights:**

**What are the main findings?**

- Successful interception exhibits within-trial, speed-dependent shifts between predictive and reactive control in both gaze and manual trajectories.
- Target occlusion reduces predictive alignment, whereas cursor occlusion has a limited impact, indicating strong reliance on internal state estimation.
**What are the implications of the main findings?**
- Visuomotor interception is governed by continuous reweighting of anticipatory and feedback-driven control, not by a fixed predictive strategy.
- Gaze and manual control show partial dissociation in their predictive dynamics, supporting distributed and effector-specific contributions to sensorimotor control.

**Abstract:**

**Background/Objectives:** Successful interception of moving targets requires combining predictive control, which anticipates future target states, and reactive control, which compensates for ongoing sensory discrepancies. How these components evolve over time and are distributed across gaze and manual behavior remains unclear. We aimed to explore the time-resolved dynamics of predictive control during continuous interception and to dissociate eye and hand contributions. **Methods:** Human participants intercepted a moving target in a two-dimensional arena using a joystick while eye movements were recorded. Target speed was systematically varied, and visual information was selectively reduced by occluding either the target or the user-controlled cursor. Predictive control was assessed using two complementary metrics: a geometric strategy index capturing moment-to-moment spatial lead or lag relative to target motion, applied separately to gaze and manual trajectories, and root mean square error (RMSE) computed relative to current and forward-shifted target positions to quantify predictive alignment. **Results:** Successful interception was characterized by structured, speed-dependent transitions between predictive and reactive control rather than a fixed strategy. Predictive alignment emerged early and was dynamically reweighted as temporal constraints increased. Gaze and manual behavior showed complementary but partially dissociable predictive signatures. Occluding the target decreased predictive alignment, whereas occluding the user-controlled cursor had comparatively minor effects, indicating strong reliance on internal state estimation rather than continuous visual feedback of the effector. **Conclusions:** Predictive and reactive control are continuously and dynamically reweighted during interception. Their interaction unfolds within single trials and depends on target dynamics and sensory availability. These findings provide quantitative evidence for time-resolved coordination between anticipatory and feedback-driven control mechanisms in goal-directed behavior.

## 1. Introduction

Intercepting a moving object is a fundamental sensorimotor capacity that requires combining sensory information with internal predictions and motor commands under strict temporal constraints. Humans rely on this ability in everyday actions such as catching objects, driving, or avoiding obstacles, making visuomotor interception a canonical model for studying sensorimotor control.

Because neural transmission across sensory pathways, synaptic relays, and motor circuits introduces inherent delays, successful interception cannot rely exclusively on reactive feedback control. Instead, it must incorporate predictive mechanisms that ‘extrapolate’ future target states to compensate for sensorimotor delays [1,2]. At the same time, purely predictive control is insufficient in dynamic environments where target motion may be uncertain or change abruptly, requiring continuous feedback-based correction [3,4,5,6]. Visuomotor interception, therefore, provides a powerful framework for examining how predictive and reactive control processes are coordinated in real time.

Predictive control is commonly associated with internal forward models that estimate future sensory consequences of action based on motion cues and physical constraints, enabling anticipatory planning despite delays [2,7,8]. Reactive control, in contrast, relies on ongoing sensory feedback to update movements online, ensuring flexibility when predictions are inaccurate or when target motion is unpredictable [4,5,9]. Although each component has been extensively studied in relative isolation, their combined operation within a single, continuous interception task remains less systematically characterized.

A substantial body of work shows that humans exploit visual motion information, such as target velocity, time-to-contact, and directional stability, to anticipate target trajectories during interception [10,11,12]. Eye movements play a critical role in this process by stabilizing visual input and sampling task-relevant information, often preceding and guiding manual responses [13,14]. At the neural level, predictive control has been linked to forward-model mechanisms involving cerebellar, parietal, and frontal circuits that estimate future sensory states [8,15]. Together, these findings establish prediction as a central component of interception behavior.

Predictive and reactive processes are increasingly viewed not as separate strategies but as interacting components of a unified control architecture whose relative contributions depend on task demands, sensory uncertainty, and effector-specific constraints [16,17]. For example, gaze behavior can anticipate target motion even when manual responses remain more feedback-driven, suggesting a potential temporal dissociation between oculomotor and limb control systems [11,13]. This functional complementarity implies that gaze may support anticipatory guidance, while manual control refines movement through ongoing feedback [18,19]. Yet, several issues and key questions remain open. First, since interception unfolds continuously over time, predictive and reactive control are often inferred indirectly from endpoint accuracy, movement onset, or averaged lead–lag measures. Such approaches obscure potential moment-to-moment transitions between anticipatory and feedback-driven control within individual trials [9,14,17]. Second, the relative contributions of gaze and manual control to predictive interception are not well dissociated. Eye–hand coordination is frequently assumed to reflect a unified predictive process, despite evidence that oculomotor and limb systems may rely on partially independent control loops with distinct temporal and sensory constraints [8,20]. Third, the role of sensory availability in sustaining predictive control remains unclear. While visual information about target motion is critical, the relative importance of visual feedback about the effector versus the target has not been systematically contrasted within a single interception framework [7,18].

Contemporary theories of sensorimotor control, including predictive processing and active inference, propose that the nervous system continuously generates predictions of sensory outcomes and acts to minimize resulting prediction errors [18,21]. Within this framework, interception emerges from a dynamic balance between anticipatory and feedback-based mechanisms. However, empirical methods capable of quantifying this balance with high temporal resolution remain scarce, and traditional endpoint-based analyses fail to capture the evolving structure of control during ongoing behavior.

The present study addresses these gaps by asking how predictive and reactive control components evolve dynamically during visuomotor interception, and how they are distributed across gaze and manual behavior under varying sensory and kinematic constraints. We designed a continuous two-dimensional interception task in which participants used a joystick to intercept a moving target while eye movements were recorded concurrently. Target speed was manipulated to vary temporal demands, and visual availability was selectively removed by occluding either the target or the user-controlled cursor at controlled distances.

We tested whether successful interception was associated with anticipatory alignment between gaze, effector, and the target’s future position, and whether these predictive signatures degrade when visual information is disrupted. To quantify these dynamics, we first derived a time-resolved geometric Strategy Index (SI) that captures moment-to-moment transitions between predictive and reactive control based on spatial lead or lag relative to the target trajectory. This index was computed separately for gaze (SI_1_) and manual behavior (SI_2_), enabling effector-specific characterization of control regimes. Complementarily, we computed the root mean square error (RMSE), which quantifies spatial deviation between participant and target trajectories relative to both current and future target positions. These measures capture distinct but related aspects of interception performance and therefore provide complementary evidence regarding predictive alignment and reactive correction.

## 2. Materials and Methods

### 2.1. Participants

The study was conducted during the first semester of 2023 using a convenience sampling strategy. Eligibility required participants to be right-handed, have normal or corrected-to-normal vision (verified with a Snellen test), and report no history of psychiatric, neurological, or neurodevelopmental disorders. Left-handed individuals were excluded to ensure consistent motor performance estimates using a joystick designed for right-handed users. All participants provided written informed consent, and participation was voluntary and uncompensated. The investigations adhered to the principles of the Declaration of Helsinki (1975, revised 2013) and received prior approval from the Ethics Committee of the Instituto de Neurociencias, Universidad de Guadalajara (protocols ET102021-330 and ET122023-382). The final sample consisted of 53 right-handed adults (27 women), aged 18–29 years (mean ± SEM: 21.6 ± 0.2 years; mode = 22 years). A within-subjects design was employed to examine visuomotor interception across variations in target speed (*v_T_*), or visual feedback. Accordingly, participants were divided into two independent cohorts. Group E_1_ (*n* = 33) completed 900 trials spanning permuted *v_T_* values from 10°/s to 60°/s in 10°/s increments, with angular range (AR) fixed at ±75° and a fixed-direction interval (FDI) of 500 ms [22]. This group was used to characterize speed-dependent dynamics within participants. Group E_2_ (*n* = 20) completed 800 trials under different degrees of visual occlusion, in which either the moving target or the joystick-controlled cursor was masked. In this condition, task parameters were held constant (*v_T_* = 30°/s, AR = ±75°, FDI = 500 ms), allowing isolation of masking effects within participants at a fixed *v_T_*. The two groups were analyzed separately, and no direct statistical comparisons were made between them. Time-resolved Strategy Index (SI) analyses were conducted only for Group E_1_, which examined speed-dependent interception dynamics. Group E_2_ was designed to evaluate the effects of visual occlusion and was therefore analyzed using RMSE-based trajectory metrics rather than SI traces. Supplementary Tables S1 and S2 report the percentage of successful interceptions for each condition in E_1_ and E_2_, respectively.

### 2.2. Visuomotor Task and Calibration Protocols

**Figure 1:**
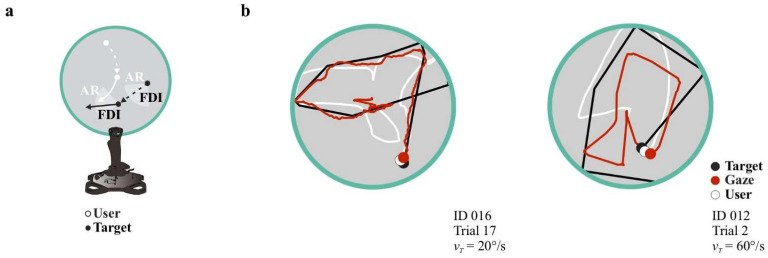
Visuomotor interception task and representative trajectories. (**a**) Task schematic. Participants used a joystick to control a white cursor and intercept a computer-controlled target (black) within a circular arena presented on a 50% gray background. Target trajectories varied in angular range (AR, ±75°) every fixed-direction interval (FDI = 500 ms). Colors correspond to the original stimulus display (user = white; target = black). (**b**) Representative trajectories from randomly selected participants. The target trajectory is shown in black, the joystick-controlled cursor in white, and gaze position in wine-red. The user is depicted as a white circle; a wine-red circle marks gaze position. All trajectories are displayed within the circular arena on a gray background. A color legend identifying target, gaze, and user markers is shown on the right.

Participants performed a visuomotor interception task in which they used a joystick to control a white dot (the user cursor) to intercept a black dot (the target) moving within a circular arena displayed at the center of a 27-inch monitor (1920 × 1080 pixels, 60 Hz; Figure 1a). Both dots were presented on a 50% gray background and had a size of 0.3° of visual angle. Participants were seated approximately 70 cm from the screen. On each trial, the user-controlled dot always started at the arena center, while the target appeared at a random location along the perimeter and moved at a constant speed (10–60°/s). The joystick had a linear gain, such that its tilt angle directly determined the cursor’s position and velocity, enabling participants to pursue and intercept the moving target.

Target motion followed piecewise linear trajectories, with direction changes occurring at fixed-direction intervals (FDI = 500 ms; sample traces in Figure 1b). At each interval, a new direction was randomly drawn from a uniform distribution within a fixed AR = ±75°. If the target encountered a boundary before completing the interval, it rebounded according to the law of reflection [23]. Each trial ended either when the participant successfully intercepted the target (i.e., a collision of the user’s and target dots) or after 5 s without interception. Although direction updates occurred at 500 ms intervals, participants did not time their interceptions to these moments. Instead, collisions occurred variably throughout the 5 s window, consistent with continuous monitoring of target motion and online adjustment of chasing strategies, rather than discrete synchronization with FDI boundaries [22,24]. Trial outcomes were indicated by auditory feedback [24].

To manipulate visual information, we introduced occlusion once a specific user-to-target distance (UTD) was reached. Thus, upon reaching a predefined UTD threshold, the dot’s contrast was set to 0%, rendering it invisible. The dot continued to move with identical kinematics. No explicit cue signaled the onset of occlusion. Occlusion distances (0°, 0.8°, 1.4°, and 2.7° of visual angle) were randomly interleaved across trials. In target occlusion conditions, participants lost sight of the moving target and had to rely on predictive strategies to complete the interception. In user-dot occlusion conditions, participants lost visual feedback of their own movement. They thus relied on proprioceptive and efference-based estimates of their cursor position relative to the visible target. These manipulations allowed us to dissociate the contributions of visual feedback from self-generated and external sensory information during interception. Although the task was two-dimensional, the stimulus–response contingencies preserved key informational variables for interception (such as *v_T_* and time-to-contact; [10,25]). Experimental sessions were designed to last less than one hour to maintain participant engagement and ensure optimal performance [23]. Each session consisted of a maximum of 900 trials, organized into three blocks separated by two five-minute resting periods. These scheduled breaks were implemented to minimize fatigue. Calibration routines were performed before each block to ensure consistent, precise data collection throughout the experiment.

### 2.3. Gaze Tracking

Binocular eye movements were recorded at 60 Hz using a Tobii Pro Fusion eye tracker (Tobii AB, Stockholm, Sweden) positioned below the monitor. Participants’ heads were stabilized with a chin and forehead rest, and ambient lighting was maintained at ~100 lux. Calibration followed a four-step procedure to ensure both spatial accuracy and physiological validity [26,27]: (1) 4-point calibration at the screen corners; (2) a custom perimeter fixation task, where participants fixated on white dots (0.6° visual angle) presented along the arena boundary; (3) a smooth pursuit test, in which participants tracked a dot moving at 10°/s horizontally and vertically; (4) a pupillary light reflex assessment, measured during alternating black–white full-screen presentations (six 35 s epochs with multiple cycles, repeated four times). Our four-step calibration and validation protocol ensured stable gaze accuracy and precision across sessions, supporting the reliability of gaze-based metrics as indices of visuomotor alignment (see Supplementary Figure S1) [24,28].

### 2.4. Data Analysis

Joystick and gaze positions were sampled at 60 Hz and analyzed in MATLAB R2023a. Raw gaze data were first inspected for signal loss and missing samples. Short gaps (≤150 ms) were linearly interpolated, whereas longer segments were excluded. Blinks were defined as periods of missing gaze or pupil data. After interpolation, gaze coordinates were transformed into polar coordinates relative to the arena center to ensure spatial alignment with target and cursor trajectories. Instantaneous gaze velocity was computed from consecutive samples without additional smoothing. Samples exceeding 100°/s were treated as outliers and removed [23]. From the cleaned gaze signal, we derived gaze-to-target distance (GTD), defined as the Euclidean distance between gaze and target positions, and gaze velocity (v_G_), used to quantify speed matching relative to the moving target. From joystick trajectories, we computed user-to-target distance (UTD), defined as the Euclidean distance between the cursor and the target, and user speed (v_U_). Together, UTD, GTD, v_U_, and v_G_ provided spatial and temporal indices of tracking accuracy and visuomotor alignment during interception [24].

To quantify rapid, frame-by-frame transitions between predictive and reactive control, we computed a strategy index based on the spatial relationship between participant behavior and target motion. Separate indices were derived for gaze (SI_1_) and joystick (SI_2_) movements. At each time point, the index was defined as the projection of the participant–target offset vector onto the target’s velocity vector:

(1) $$SI\left(t\right)=\left(x_{user}\left(t\right)-x_{target}\left(t\right)\right)\cdot \frac{v_{T}\left(t\right)}{\left\|v_{T}\left(t\right)\right\|}$$

**Figure 2:**
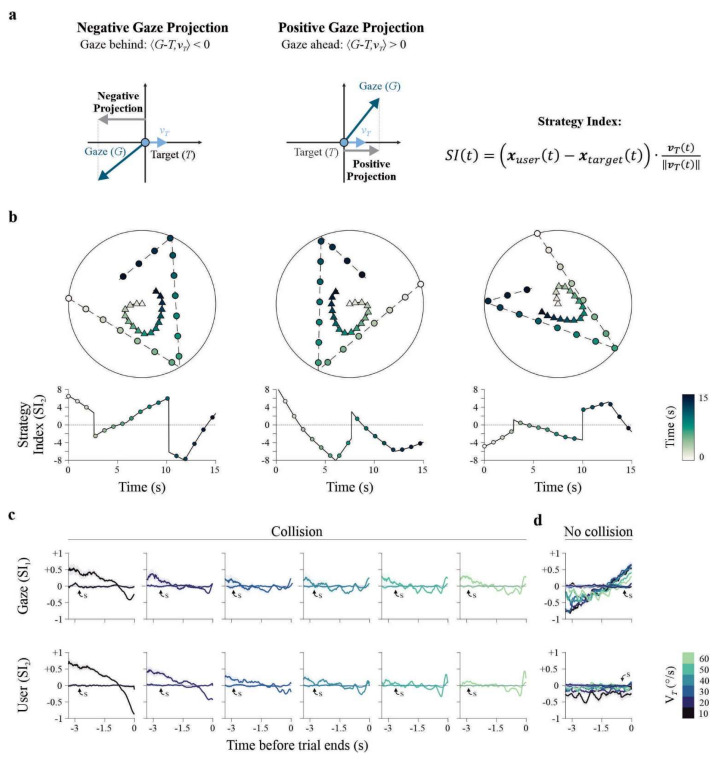
Strategy index dynamics during visuomotor interception. (**a**) Illustration of the strategy index computation. The spatial offset between participant (gaze or joystick) and target positions is projected onto the target’s velocity vector. Negative values indicate reactive pursuit (spatial lag), whereas positive values indicate predictive alignment (spatial lead). (**b**) Simulated interception trajectories (target: triangles; participant: circles) illustrating how spatial alignment and velocity direction generate informative strategy index values. Continuous trajectories show interception dynamics; color encodes elapsed time. Negative values reflect lagging behind the target, whereas positive values reflect leading along its future path. (**c**) Successful trials. Event-averaged strategy index traces for gaze (upper panels, SI_1_) and joystick movements (lower panels, SI_2_), aligned to the interception moment (time 0) and color-coded by *v_T_* (*v_T_* = 10–60°/s). Traces illustrate an early predictive regime, a transition toward reactive tracking, and a late return to predictive alignment before collision. For each *v_T_*, we also plot the corresponding average surrogate trace; these are labeled with “s” in each panel, with arrows indicating the surrogate mean trajectory. (**d**) Unsuccessful trials. Event-averaged gaze and user indices aligned to trial end (time 0). The structured predictive–reactive–predictive sequence observed in successful interceptions is absent. As in (**c**), average surrogate traces are shown for each *v_T_* and marked with “s” and arrows. Data from Group E_1_ (*n* = 33 participants, 900 trials each; FDI = 500 ms).

Positive values indicate predictive alignment, where gaze or movement is spatially ahead of the target along its direction of motion; negative values indicate reactive pursuit, trailing the target’s current position (Figure 2a). Traces were event-aligned to interception for successful trials or to trial termination for unsuccessful trials, enabling high-temporal-resolution analyses of dynamic strategy shifts [24].

To illustrate the geometric properties of the strategy index, we implemented a simple numerical simulation of interception in a virtual circular arena. The target moved at constant speed within a two-dimensional circular arena, rebounding at the boundary according to specular reflection [22]. Target velocity was estimated numerically from position traces. The simulated user was initialized at the arena center and generated smooth trajectories via a first-order velocity update rule. At each time point, the strategy index was computed using Equation (1). Its sign depended solely on the relative spatial configuration of the user and target, independent of specific controller parameters (Figure 2b).

To verify that SI_1_ and SI_2_ captured predictive alignment rather than artifacts of movement speed or generic kinematic structure, we compared the empirical traces against a kinematically matched null model. For each trial, we generated surrogate gaze and joystick-movement trajectories using a temporally shuffled correlated random walk. This procedure extracted the instantaneous step distances (i.e., speeds) and relative turn angles from each participant’s original trajectory and randomly permuted their temporal order. The shuffled steps were then sequentially reapplied from the true initial position, producing a surrogate path that preserved the empirical distributions of speed and turning angles while eliminating their original temporal structure. This manipulation preserved low-level kinematic statistics while eliminating temporal coupling between participant movement and target motion. Representative examples from three participants, illustrating kinematic distributions alongside spatial divergence from the true target-coupled paths, are shown in Supplementary Figure S2.

To assess average predictive alignment in gaze and manual behavior, we computed the root mean square error (RMSE) between participant trajectories (gaze or cursor) and the target’s trajectory. RMSE provided a continuous, unsigned measure of spatial deviation and was computed as:

(2) $$RMSE=\sqrt{\frac{1}{N}\sum_{i=1}^{n}{\left(P_{i}-T_{j}\right)}^{2}}$$

where *P_i_* denotes the participant’s position (gaze or cursor) at frame *i*, and *T_j_* represents the target position at frame *j* = *i* + *k*. The current-alignment condition corresponds to *k* = 0, whereas forward-alignment values were computed across a window extending up to 100 frames ahead (~1666 ms). For statistical testing, we compared two specific offsets within each participant: RMSE at the current frame (*k* = 0) and RMSE at the maximum forward shift (*k* = 100 frames). Paired statistical tests therefore assessed whether trajectories were spatially closer to the target’s future position than to its instantaneous position. RMSE traces shown in the figures display the full offset range (0–100 frames), allowing visualization of where the minimal error occurs along the forward axis, but statistical inference is based on the comparison between the current and the 100-frame forward conditions. Smaller RMSE values at positive offsets indicate closer alignment with future target positions and were operationally interpreted as evidence of predictive behavior; larger values would reflect less predictive, more reactive tracking [4,23].

RMSE computations terminated before trial completion due to the absence of future target data beyond the forward window (i.e., the RMSE traces were truncated prior to interception). For clarity, RMSE values are plotted as a function of forward temporal offset (in milliseconds). RMSE was computed separately for user and gaze positions (spatial prediction) and for *v_U_* and *v_G_* (velocity alignment), allowing us to dissociate positional anticipation from speed matching. We chose RMSE as a ‘summary metric’ of interception performance because it integrates spatial deviations over the full trajectory rather than relying on single events, yielding stable estimates of spatial coupling across trials [29,30]. Comparing RMSE between current and future target positions provided an indirect but informative index of predictive alignment in both gaze and manual control [2,11,16,31].

### 2.5. Statistical Analysis

We determined the sample size based on pilot data showing large effects for paired comparisons of gaze RMSE. An a priori power analysis conducted in G*Power 3 indicated that a minimum of 15 participants were required to achieve 95% statistical power at an *α* level of 0.05. Data preprocessing and analyses were conducted in MATLAB R2023a. To examine associations between visuomotor parameters, we computed Pearson correlation coefficients (*r*) and fitted linear regression models to test whether *v_T_* influenced: (i) the timing of strategy index shifts and (ii) gaze–hand correlations, reporting the regression slope (*m*) and associated *p*-values. We assessed data normality using the Shapiro–Wilk test. For normally distributed data, we applied paired-sample *t*-tests to compare RMSE values between current and future target positions, reporting the *t*-value (*df*), *p*-value, and Cohen’s *d* as the effect size. When normality was violated, we used Wilcoxon signed-rank tests, reporting the *Z* statistic and *p*-value. All statistical tests were two-tailed, and significance was defined as *p* < 0.05 (or *q* < 0.05 after correction). To prevent inflated Type I error rates, we applied Bonferroni correction for pairwise post hoc tests and false discovery rate (FDR) adjustment when evaluating multiple dependent variables. We performed permutation tests (1000 iterations) to validate differences in RMSE between the current and forward frames against null distributions. To examine the effects of visual masking, we conducted repeated-measures ANOVAs comparing RMSE metrics (user position, gaze position, user velocity, gaze velocity) across masking distances (0°, 0.8°, 1.4°, 2.7°) at both the current frame and a forward frame 100 samples (~1666 ms) ahead. When main effects were detected, Bonferroni-corrected pairwise comparisons identified the specific distances that differed. Effect sizes for these analyses are reported as generalized eta squared (*η*^2^*G*). All statistical analyses were performed on trial-averaged metrics computed within each participant, and group-level inferences were based on these participant means. Successful (collision) and unsuccessful (no-collision) trials were analyzed separately, as prior work indicates that they reflect qualitatively distinct visuomotor regimes [22,24,32]. Treating them independently avoids conflating divergent behavioral dynamics and allows clearer interpretation of predictive versus reactive control signatures. Unless otherwise specified, results are reported as mean ± standard error of the mean (SEM).

## 3. Results

### 3.1. Speed-Dependent Visuomotor Coordination

Participants performed a visuomotor interception task in which they used a joystick to control a cursor and intercept a moving target (Figure 1a). Target speed (*v_T_*) ranged from 10°/s to 60°/s and was randomly permuted across trials. To characterize how visuomotor behavior scaled with task demands, we quantified user speed (*v_U_*), gaze speed (*v_G_*), and spatial distances between gaze, user, and target. Representative single-trial trajectories from two randomly selected participants are shown in Figure 1b. Interception performance remained relatively constant throughout the experimental sessions. We assessed stability using interception success rates and average collision times, quantified across non-overlapping trial blocks. Linear regressions revealed no significant performance drift in either experimental group (E_1_ and E_2_, both *p* ≥ 0.13), and median-split comparisons between the first and second halves of the session showed no differences (paired *t*-tests: both *p* ≥ 0.48; Supplementary Figure S3).

We first assessed the dependency of *v_U_* and *v_G_* on the task’s independent parameters, irrespective of interception outcome. Both *v_U_* and *v_G_* exhibited a similar, non-linear dependence on *v_T_* (Supplementary Figure S4). At low *v_T_*, both measures exceeded *v_T_*; near intermediate speeds (~50°/s), *v_U_*, and *v_G_*, converted towards *v_T_*; and at higher speeds, both *v_U_* and *v_G_* systematically undershot *v_T_*. These relationships were computed from event-averaged traces aligned to trial onset, capturing the early sensorimotor response to target motion. Interestingly, temporal analyses revealed rapid adjustment of *v_U_* toward *v_T_* within approximately 650 ms after trial onset, as reflected by an early peak in the time derivative of *v_U_* (Supplementary Figure S4a, lower panel). This fast convergence indicates efficient scaling of motor output to *v_T_*. To examine outcome-dependent effects, we analyzed event-averaged traces aligned to collision (successful trials) or trial termination (no-collision trials). During the final 800 ms of the trial, both speed and spatial measures diverged across outcomes: in successful trials, *v_U_*, *v_G_*, and spatial performance metrics (UTD, GTD) converged toward the target, whereas in unsuccessful trials these measures remained more elevated (Supplementary Figure S4b,c).

To assess the contribution of visual inputs, we manipulated visual availability by masking either the user-controlled dot or the target at multiple user-to-target distances. Masking the user-controlled dot had limited impact on interception performance, whereas masking the target strongly disrupted behavior, increasing spatial error and reducing speed alignment (Supplementary Figure S5). A Friedman test confirmed a strong main effect of masking condition, with target masking producing greater impairment than user masking across all distances (χ^2^_(2)_ = 813.75, *p* < 0.001; all post hoc *p* < 0.001).

### 3.2. Strategy Indices Reveal Dynamic Transitions Between Predictive and Reactive Control

The continuous nature of the interception task, with uninterrupted target, gaze, and manual trajectories, enables frame-by-frame analysis of full positional dynamics. This temporal resolution allows predictive and reactive components to be dissociated based on their relative timing. Predictive control is expected to manifest as spatial alignment that precedes target motion, whereas reactive control should appear as compensatory adjustments following positional error signals. In classical negative-feedback systems, errors temporally precede corrective motor commands; therefore, the temporal coupling between error dynamics and movement velocity provides a functional signature of feedback regulation.

Several studies have operationalized this framework using continuous geometric relationships between agent and target. Lead–lag analyses of signed distance errors have demonstrated predictive control when changes in spatial separation precede motor output [33]. Directional alignment metrics, such as the cosine between pursuit and target velocity vectors, have revealed anticipatory motor responses on the order of ~150 ms [14]. Other approaches have emphasized angular geometry, including constant-bearing or target-heading formulations, to capture prediction in interception tasks [10,34]. Together, these approaches converge on the idea that prediction can be inferred from systematic spatial alignment with future target states.

Building on this logic, we defined a Strategy Index (SI) to quantify predictive and reactive alignment continuously at single-frame resolution using only the spatial relationship between participant behavior and target motion. Unlike averaged measures of speed or distance, which reflect raw performance magnitude, the SI directly indexes whether behavior is aligned with the target’s current or future trajectory. Operationally, the SI was computed as the signed projection of the participant–target offset onto the target’s velocity vector, yielding a geometric measure of spatial lead (positive values) or lag (negative values) relative to target motion (Figure 2a).

To initially characterize the geometric behavior of the Strategy Index (SI), we conducted numerical simulations in which user-related parameters were varied while target dynamics were held constant. We aimed to generate controlled scenarios in which the participant trajectory systematically led or lagged the target, thereby producing a continuum from reactive to predictive alignment relative to the target’s velocity vector (Figure 2b). Note that such leading and lagging configurations can also arise transiently in real trials due to task contingencies (e.g., rebounds or abrupt directional changes) and therefore should not be overinterpreted at the single-trial level. For this reason, SI was analyzed as a time-resolved metric and subsequently averaged across large numbers of trials and participants, allowing incidental geometric fluctuations to cancel out while preserving consistent population-level patterns, if present.

We computed SI separately for gaze (SI_1_) and joystick trajectories (SI_2_). Event-averaged traces (Figure 2c,d) were aligned to the end of each trial: for collision trials, alignment was to the moment of interception; for no-collision trials, to trial termination. Thus, time zero corresponds to trial end and is plotted at the rightmost edge of each trace. In successful trials, both gaze and manual indices exhibited a structured temporal evolution. An early period of positive SI (spatial lead) was followed by a transition toward negative values (relative lag), and then by a late return toward positive SI immediately preceding interception. The timing of the predictive-to-reactive transition shifted systematically with *v_T_*. For gaze, the zero-crossing occurred progressively earlier as *v_T_* increased (from ~817 ms at 10°/s to ~2200 ms at 50°/s; linear trend: *m* = 27.70, *p* = 0.031; Figure 2c, upper panels). Joystick indices showed a comparable speed-dependent shift (crossings ~817–1983 ms; *m* = 23.80, *p* < 0.001; Figure 2c, lower panels). Notably, in unsuccessful trials, this temporal organization was not observed (all *p* > 0.25; Figure 2d). In this case, instead of displaying a clear ‘predictive–reactive–predictive’ sequence, SI_1_ values tended to remain negative during intervals in which successful trials showed positive alignment, and SI_2_ values were predominantly negative throughout the pre-termination period. This difference reflects altered temporal structure and alignment dynamics rather than a simple sign inversion.

To determine whether these SI dynamics reflected genuine target-related prediction rather than generic kinematic structure, we compared empirical trajectories to a kinematically matched surrogate model (see Section 2). In successful trials, both user and gaze indices were significantly higher than their respective surrogate baselines (*t*_(32)_ = 17.76, *p* < 0.001, *d* = 1.79; *t*_(32)_ = 9.92, *p* < 0.001, *d* = 1.00), indicating active spatial prediction of the target’s path. In unsuccessful trials, indices were significantly more negative than surrogate trajectories across all *v_T_* (all *p* < 0.001; *d* = −0.77 to −3.98), demonstrating systematic lag relative to the target rather than random or passive movement. We provide a detailed frame-by-frame comparison across velocities in Supplementary Figure S6.

**Figure 3:**
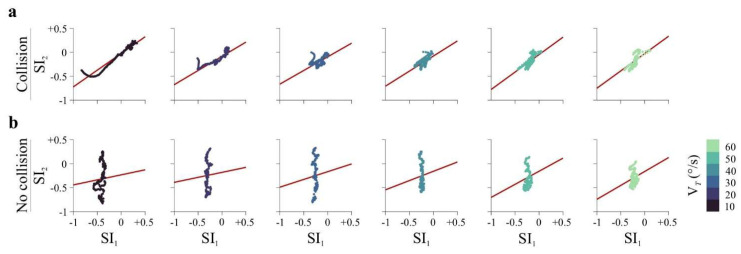
Gaze–hand coordination during interception. Participant-level coupling between gaze (SI_1_) and joystick (SI_2_) strategy indices within the pre-interception time window. For each participant and target velocity (*v_T_*), we computed the Pearson correlation (*r*) and regression slope between SI_1_ and SI_2_ across frames. Correlation coefficients were Fisher Z-transformed prior to group-level statistical testing. In the scatter panels, individual data points correspond to participant-level values and are displayed after sorting observations by SI_1_ magnitude (from smallest to largest). This ordering is applied solely for visualization purposes, producing the apparent monotonic arrangement of points along the *x*-axis without affecting the underlying statistical analyses. (**a**) Successful (collision) trials. Gaze and hand strategies were positively coupled across all *v_T_*, with average correlations ranging from 0.48 to 0.69 (all *p* < 0.001). (**b**) Unsuccessful (no-collision) trials. Positive gaze–hand coupling was also observed, although weaker (average *r* = 0.15–0.45; all *p* < 0.05).

Finally, we assessed whether predictive strategies in gaze and hand movements covaried (Figure 3). To avoid biases associated with autocorrelated time-series data, we first computed participant-level summary statistics within the pre-interception time window. For each participant and *v_T_*, we calculated the Pearson correlation (*r*) and regression slope between SI_1_ and SI_2_. Correlation coefficients were then Fisher Z-transformed prior to group-level testing. Across all *v_T_*, gaze and hand strategies were positively coupled. In successful trials, average correlations ranged from 0.48 to 0.69 (all *p* < 0.001), whereas in unsuccessful trials they ranged from 0.15 to 0.45 (all *p* < 0.05). To test whether this coupling scaled with task demands, we fitted a linear mixed-effects model with *v_T_* as a fixed effect and participant as a random intercept. *v_T_* modulated the gaze–hand regression slope in both successful (*t*_(196)_ = 2.38, *p* = 0.017; Figure 3a) and unsuccessful trials (*t*_(196)_ = 8.68, *p* < 0.001; Figure 3b), indicating that the strength of gaze–hand coupling varies with movement speed.

### 3.3. Predictive Components in User and Gaze Trajectories During Interception

The preceding analyses revealed that successful interception is characterized by structured, speed-dependent transitions between predictive and reactive control, captured by time-resolved strategy indices. To complement these dynamic measures, we used root mean square error (RMSE) to assess whether predictive alignment is evident at the level of overall trajectory geometry.

In the present task, RMSE provides a global measure of performance by quantifying the average spatial deviation between user or gaze trajectories and the target across the full trajectory preceding interception. Importantly, this includes pursuit periods, directional changes, and rebound events within the arena. As such, RMSE does not resolve moment-to-moment strategy shifts but instead summarizes how closely behavior aligns with the target over extended portions of the trial. We therefore used RMSE to assess whether, on average, trajectories were better aligned with the target’s current position or with its future position, thereby providing an aggregate index of predictive versus reactive control.

**Figure 4:**
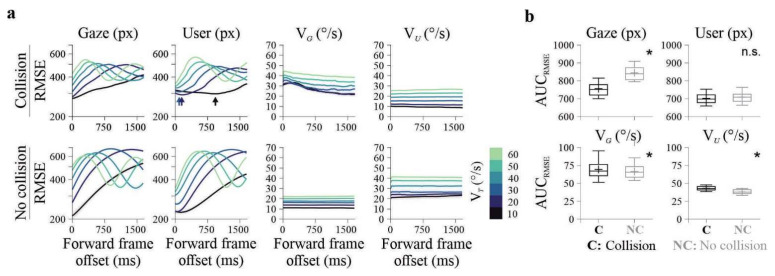
Predictive alignment quantified by RMSE metrics. (**a**) Root mean square error (RMSE) for gaze position, user position, gaze velocity and user velocity, across *v_T_* (*v_T_* = 10–60°/s; color-coded). Upper panels show collision trials; lower panels show no-collision trials. Positional RMSE is plotted on a logarithmic scale (pixels). Arrows indicate local minima where RMSE relative to future target positions was lower than RMSE relative to current positions, reflecting optimal predictive alignment. Visual angle conversion: 39.24 px/° (70 cm viewing distance). (**b**) Area under the curve (AUC) of RMSE traces for the four metrics, comparing collision (black) and no-collision (gray) trials. Error bars indicate ±SEM. Asterisks denote statistically significant differences after correction for multiple comparisons. Data from Group E_1_ (*n* = 33 participants, 900 trials each).

For each *v_T_*, RMSE was computed relative to the target’s instantaneous position and to a forward-shifted position (100 frames ahead, ~1666 ms). For statistical testing, we compared RMSE at the current frame (k = 0) with RMSE at the maximum forward offset (*k* = 100 frames). The RMSE curves plotted in Figure 4 show the full range of temporal offsets (0–100 frames) to illustrate how alignment evolves as the target trajectory is shifted forward in time. Arrows marking minima therefore indicate the offset at which RMSE reached its lowest value within this forward window and are provided for visualization; statistical significance is based on the paired comparison between the current and 100-frame forward conditions. A predictive component was operationally defined as a reduction in RMSE when referenced to the future position compared with the current one (i.e., reflecting closer spatial alignment with the target’s upcoming state). Such reductions provide strong evidence of anticipatory positioning. In contrast, equal or larger RMSE values for forward positions do not uniquely indicate the absence of prediction. Increased error relative to future positions may arise from reactive corrections, task-specific constraints (e.g., rebounds or abrupt directional changes), or limited detectability of upcoming target states. For velocity traces, smaller RMSE values reflect closer speed matching between participant and target but do not necessarily imply anticipatory speed advantage.

In successful (collision) trials, user position RMSE revealed reductions at positive temporal offsets, consistent with predictive alignment at lower target velocities. Forward-shifted RMSE values were smaller than current-position RMSE at 10°/s (*t*_(32)_ = 9.56, *p* < 0.001, *d* = 0.97, *q* = 0.002), 20°/s (*t*_(32)_ = 19.7, *p* < 0.001, *d* = 1.99, *q* = 0.002), 30°/s (*t*_(32)_ = 13.7, *p* < 0.001, *d* = 1.39, *q* = 0.002), and 40°/s (*t*_(32)_ = 2.25, *p* = 0.013, *d* = 0.23, *q* = 0.019; Figure 4a). No such predictive reductions were detected at higher speeds (50–60°/s; all *q* = 1). Similarly, gaze position RMSE showed no forward-offset reductions at any *v_T_* (all *q* > 0.05). Velocity-based RMSE metrics did not show consistent reductions at forward temporal offsets. Neither user nor gaze velocity RMSE exhibited a clear or systematic decrease when the target trajectory was shifted forward in time, and this pattern held across both successful and unsuccessful trials (all *q* > 0.05; Figure 4a).

We also tested whether the qualitative differences in RMSE traces between successful and unsuccessful trials could distinguish outcomes. We computed the area under the curve (AUC) for each RMSE trace on a per-trial basis, averaged within participants, and compared collision and no-collision trials at the group level (Figure 4b). For user position RMSE, AUCs did not differ between outcomes (*t*_(32)_ = −1.87, *p* = 0.065). By contrast, gaze position RMSE exhibited lower overall trajectory error (AUC) in successful trials (*t*_(32)_ = −20.1, *p* = 0.001). Velocity measures showed the opposite pattern: both user velocity (*t*_(32)_ = 16.3, *p* = 0.001), and gaze velocity (*t*_(32)_ = 3.05, *p* = 0.003) exhibited larger AUCs in successful collision trials.

### 3.4. Impact of Visual Occlusion on RMSE Metrics

We asked whether predictive alignment, as indexed by RMSE, was preserved when visual information was selectively removed. Visual masking was applied either to the user-controlled dot or to the target at four user-to-target distances (0°, 0.8°, 1.4°, and 2.7° of visual angle), randomly interleaved across trials. RMSE for user position, gaze position, and their corresponding velocities was computed relative to the target at the current frame and at a forward frame 100 samples ahead (~1666 ms). Effects of masking distance were evaluated using repeated-measures ANOVAs with Bonferroni-corrected post hoc comparisons, conducted separately for successful and unsuccessful trials.

**Figure 5:**
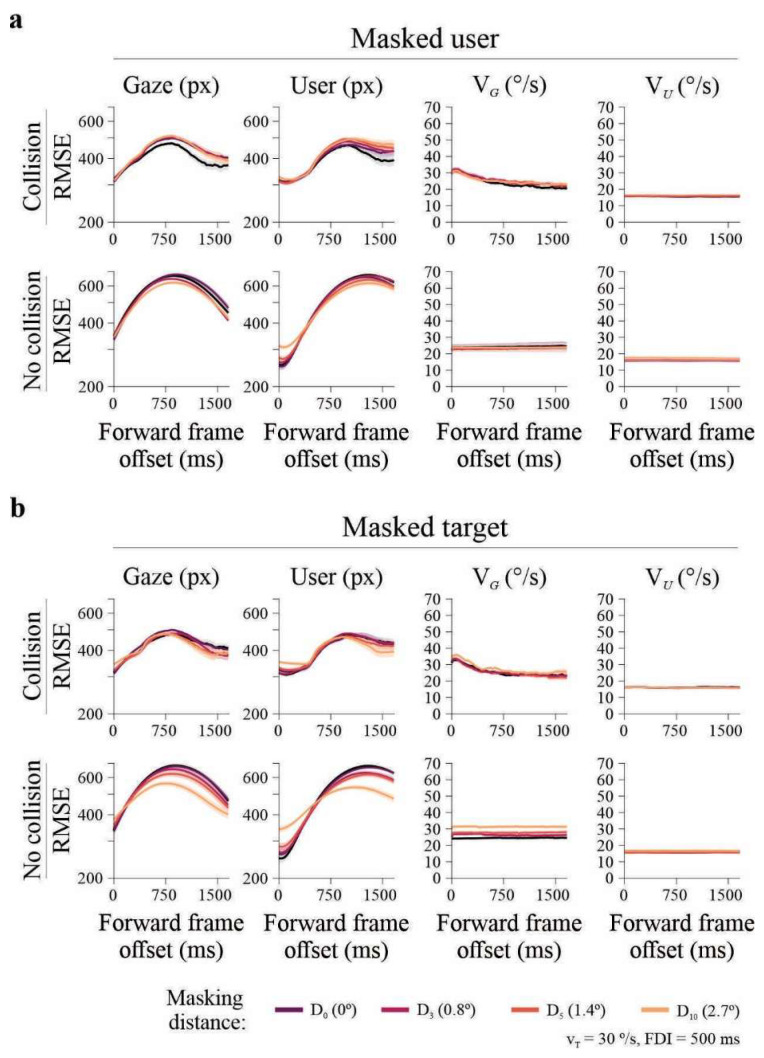
Effects of visual occlusion on RMSE metrics. (**a**) Occlusion of the user-controlled cursor at four masking distances (0°, 0.8°, 1.4°, 2.7° visual angle). RMSE for user and gaze positions is shown for collision (upper panels) and no-collision (lower panels) trials. Predictive reductions in RMSE were largely preserved, indicating maintained control despite loss of visual effector feedback. (**b**) Occlusion of the target at the same distances. Both user and gaze positional RMSE increased strongly with occlusion, particularly in no-collision trials, abolishing predictive reductions. Black line represents average RMSE computed from no-masking trials (matched in number of repetitions), extracted prior to the masking experiments. Data from Group E_2_ (*n* = 20 participants, 800 trials each).

When the user-controlled dot was masked, RMSE metrics showed only modest changes. In successful trials, user position RMSE varied across masking distances at both the current frame (*F*_3,177_ = 9.78, *p* < 0.001, *η*^2^*G* = 0.06), and the future frame (*F*_3,177_ = 9.13, *p* < 0.001, *η*^2^*G* = 0.087), driven by increased error at the largest occlusion (2.7°; all post hoc *p* < 0.01; Figure 5a). Gaze position RMSE differed across masking distances at the current frame (*F*_3,177_ = 3.05, *p* = 0.03, *η*^2^*G* = 0.021), but not at the future frame. Velocity-based RMSE showed small effects at the future frame for both user velocity (*F*_3,177_ = 2.85, *p* = 0.039), and gaze velocity (*F*_3,177_ = 3.43, *p* = 0.018), again attributable to increased error at the largest occlusion. In unsuccessful trials, only user position RMSE varied significantly with masking distance (*F*_3,24_ = 10.08, *p* < 0.001, *η*^2^*G* = 0.23).

In contrast, masking the target produced pronounced effects across multiple RMSE measures. In successful trials at the current frame, both user position RMSE (*F*_3,177_ = 52.3, *p* < 0.001, *η*^2^*G* = 0.286) and gaze position RMSE (*F*_3,177_ = 20.4, *p* < 0.001, *η*^2^*G* = 0.147; Figure 5b) increased with masking distance, with the largest errors observed at 2.7°. Gaze velocity RMSE also differed across distances (*F*_3,177_ = 4.56, *p* = 0.004, *η*^2^*G* = 0.031). Unsuccessful trials showed a comparable pattern, with a strong effect of masking distance on user position RMSE (*F*_3,24_ = 18.1, *p* < 0.001, *η*^2^*G* = 0.509), but at the forward frame, no RMSE measure showed significant effects of masking distance (all *p* > 0.017), indicating the absence of predictive alignment under target occlusion.

## 4. Discussion

We investigated how predictive and reactive visuomotor strategies evolve during continuous interception by manipulating *v_T_* and visual availability while measuring gaze and hand behavior. By combining time-resolved geometric indices with trajectory-level performance metrics, we showed that successful interception emerges from a dynamic reweighting of anticipatory and feedback-driven control rather than from a fixed strategy.

Across conditions, participants rapidly scaled gaze and manual velocities to target velocity, reaching near convergence within approximately 650 ms. This rapid adjustment indicates that velocity information remains continuously available for online control. The non-linear relation between *v_T_* and participant speed (overshooting at low velocities, matching at intermediate velocities, and undershooting at high velocities) reveals systematic adaptation to task demands. Overshooting at low velocities could compensate for sensorimotor delays, whereas undershooting at high velocities is consistent with perceptual and biomechanical constraints under time pressure [15,35,36]. Convergence at intermediate velocities suggests an operating regime (a ‘sweet-spot’) in which perceptual estimation and motor output are optimally balanced [11]. These results indicate graded parameter adjustment within trials and extend hybrid models of predictive–reactive control by quantifying their temporal structure under controlled kinematic and sensory manipulations.

Visual occlusion revealed a clear functional asymmetry. Removing visual feedback about the effector produced only minor performance costs, whereas removing visual information about the target impaired interception across spatial and velocity measures. We therefore propose that predictive control depends primarily on continuous access to target motion, while effector state can be maintained through proprioceptive and efference-based signals. This pattern aligns with models that emphasize internal-state estimation and forward prediction, supported by non-visual feedback [8,18].

The Strategy Index allowed us to resolve how alignment evolves within trials. In successful interceptions, both gaze and hand exhibited structured transitions: early predictive alignment, followed by a shift toward more reactive tracking, with transition timing scaling monotonically with target velocity. Faster targets induced earlier transitions, consistent with tighter temporal constraints. The final 150–200 ms before interception fall within the visuomotor delay window, during which new sensory input cannot modify ongoing motor commands [3,5,37]. The stabilization of strategy indices during this late phase indicates that apparent “reactive” corrections likely reflect the execution of earlier predictive motor plans rather than real-time feedback control. This pattern is consistent with forward-model architectures that compensate for sensorimotor delays by ‘extrapolating’ future states [1]. Unsuccessful trials lacked these structured transitions and remained dominated by a lagging alignment. Target occlusion abolished predictive shifts, reinforcing the conclusion that anticipatory control requires continuous sensory evidence about target motion.

Gaze and hand were subject to identical task constraints and covaried at the level of strategy. Across target velocities, their strategy indices were significantly correlated, with stronger coupling in successful than in unsuccessful trials. The regression slope increased with target velocity, indicating that cross-effector coordination scaled with task demands. These findings suggest that predictive alignment in gaze and hand is coordinated rather than independent. The weaker correlations observed in unsuccessful trials point to reduced consistency in this coordination, rather than complete decoupling. Successful interception, therefore, depends not only on alignment within each effector but also on the dynamic coupling between them.

RMSE-based trajectory analyses complemented the time-resolved strategy indices by quantifying global spatial alignment. Forward-shift RMSE reductions were observed primarily for manual position, particularly at lower target velocities and in successful trials. This pattern suggests that interception at slower speeds permits sustained anticipatory alignment with future target location. In contrast, gaze trajectories did not exhibit comparable forward reductions, consistent with the possibility that oculomotor behavior relies on rapid sampling strategies that erode sustained spatial offsets. Because RMSE aggregates unsigned deviations across entire trajectories, it could capture overall alignment but would attenuate brief or transient predictive episodes. Moreover, substantial within-subject variability in visuomotor behavior [20,38,39] likely further reduces the detectability of short-lived anticipatory RMSE events at the group level.

The Strategy Index and RMSE capture distinct geometric properties of alignment. The Strategy Index preserves directionality by projecting participant–target offset onto target velocity and thus reveals temporal reweighting between predictive and reactive modes. Forward-offset RMSE measures unsigned proximity to temporally shifted target states and therefore indexes future alignment without encoding directional structure. Although reduced forward RMSE alone cannot prove anticipation, a purely lagged strategy would predict increased error when shifting the target trajectory forward in time. The observed forward-RMSE reductions are therefore inconsistent with passive delay and support the idea of a genuine spatio-temporal alignment. Divergences between SI and RMSE are expected because they isolate different dynamical aspects of interception.

Our behavioral findings are compatible with interactions among cerebellar, parietal, and frontal systems involved in predictive control. Cerebellar circuits likely support forward estimation, parietal regions integrate visual motion with efference-based signals, and premotor areas contribute to anticipatory preparation [40,41,42]. The gradual predictive–reactive transitions we observed may reflect evolving engagement of these networks as uncertainty and temporal pressure increase. Although we did not record neural activity, the temporal structure of the indices is consistent with preparatory processes associated with readiness-related potentials [43,44].

Several limitations constrain interpretation. We implemented the task in a two-dimensional joystick-controlled environment, which enabled precise quantification but reduced ecological validity relative to natural three-dimensional interception. The 60 Hz gaze sampling rate limits detection of microsaccades and fine corrective events. We computed the Strategy Index continuously without segmenting oculomotor regimes, so gaze indices reflect aggregate alignment rather than regime-specific mechanisms. The small arena and frequent target rebounds introduce abrupt directional changes that can transiently reverse index sign; large-scale event averaging mitigates this variability but does not eliminate local effects. We did not quantify prior visuomotor experience, which may shape baseline performance. Finally, we relied exclusively on behavioral and ocular measures. Future work could combine this framework with electrophysiology, neuroimaging, or perturbation approaches to establish causal links between predictive–reactive transitions and specific neural circuits.

## 5. Conclusions

We show that the predictive and reactive components of visuomotor control evolve dynamically within interception trials and depend on target velocity and sensory availability. Successful interception requires structured forward alignment of manual behavior at lower velocities and flexible reweighting as temporal constraints tighten. Removing visual access to the target reduces these predictive signatures, demonstrating that anticipation depends on sampling of continuous sensory evidence. By combining a signed geometric index with temporally shifted RMSE analyses, we provide complementary measures that dissociate directional alignment from unsigned proximity. Together, these results support a hybrid architecture in which predictive and feedback-driven processes interact continuously, and they offer a tractable behavioral framework for testing computational accounts of predictive processing and active inference in dynamic sensorimotor tasks.

## Data Availability

Raw data used for this study are openly available in https://osf.io/5yc8v/?view_only=ecc0e958caa742ffb8f365e4dfc7a764, 11 September 2025.

## Supplementary Materials

The following supporting information can be downloaded at: https://www.mdpi.com/article/10.3390/brainsci16030322/s1, Table S1: Probability of successful interception (E_1_). Table S2. Probability of successful interception (E_2_). Figure S1. Visuomotor task calibration and control procedures. Figure S2. Construction and validation of the kinematically matched surrogate null model. Figure S3. Stability of Interception Performance Across the Experimental Session (E_1_ and E_2_). Figure S4. Speed adjustment and spatial convergence during interception. Figure S5. Effects of visual occlusion on visuomotor parameters. Figure S6. Validation of gaze (SI_1_) and manual (SI_2_) strategy indices against a kinematically matched surrogate null model across target speeds.

## Abbreviations

AR	Angular Range
FDI	Fixed-Direction Interval
GTD	Gaze-to-Target Distance
RMSE	Root Mean Square Error
SI1	Strategy Index 1 (Gaze)
SI2	Strategy Index 2 (User/Joystick)
UTD	User-to-Target Distance
*v_G_*	Gaze Velocity
*v_T_*	Target Speed
*v_U_*	User Speed
