# Supplementary material for: Predictive and Reactive Control During Interception
Source: Brain Sci. 2026 Mar 18;16(3):322. doi: 10.3390/brainsci16030322 (PMC13024469; doi:10.3390/brainsci16030322)
Supplement: Supplementary file 1 [file brainsci-16-00322-s001.zip › brainsci-4166652-supplementary.pdf]

Supplemental Material for

Predictive and Reactive Control During Interception

Mario Treviño[1*], Nathaly Martín[2], Andrea Barrera[2], Inmaculada Márquez[1,3,4]

[1]Laboratorio de Plasticidad Cortical y Aprendizaje Perceptual, Instituto de Neurociencias, Universidad de Guadalajara, Guadalajara, Jalisco, México
[2]Departamento de Ciencias Médicas y de la Vida, Centro Universitario de la Ciénega, Universidad de Guadalajara, Ocotlán, México
[3]Departamento de Psicología, Centro Universitario de la Ciénega, Universidad de Guadalajara, Ocotlán, México
[4]Laboratorio de Neurofisiología, Departamento de Bioingeniería Traslacional, Centro Universitario de Ciencias Exactas e Ingenierías, Guadalajara, Jalisco, México

Correspondence:

*Dr. Mario Treviño (mario.trevino@academicos.udg.mx), Laboratorio de Plasticidad Cortical y Aprendizaje Perceptual, Instituto de Neurociencias, Universidad de Guadalajara. Francisco de Quevedo 180, Arcos Vallarta. C.P. 44130. Guadalajara, Jalisco, México.

Keywords: Visuomotor interception; Predictive processing; Reactive control; Gaze–hand coordination; Visual occlusion

Exp.	FDI (ms)	V_T (°/s)	N. Rep.	Probability of interception			Number of participants
E_1	500	10	150	96.76%	±	1.05%	33
	500	20	150	91.95%	±	1.41%	
	500	30	150	87.04%	±	1.68%	
	500	40	150	80.32%	±	2.18%	
	500	50	150	73.92%	±	2.19%	
	500	60	150	66.95%	±	2.05%	

Supplementary Table S1. Probability of successful interception (E_1).

Group-level probability of successful interception for Group E_1.

Exp.	FDI (ms)	V_T (°/s)	Masking distance (°)	N. Rep.	Probability of interception			Number of participants
E_2	500	30	0	150	91.73%	±	1.44%	20
	500	30	0.08	150	88.34%	±	1.43%	
	500	30	0.14	150	83.99%	±	1.55%	
	500	30	0.27	150	70.74%	±	2.09%	

Supplementary Table S2. Probability of successful interception (E_2).

Group-level probability of successful interception for Group E_2.

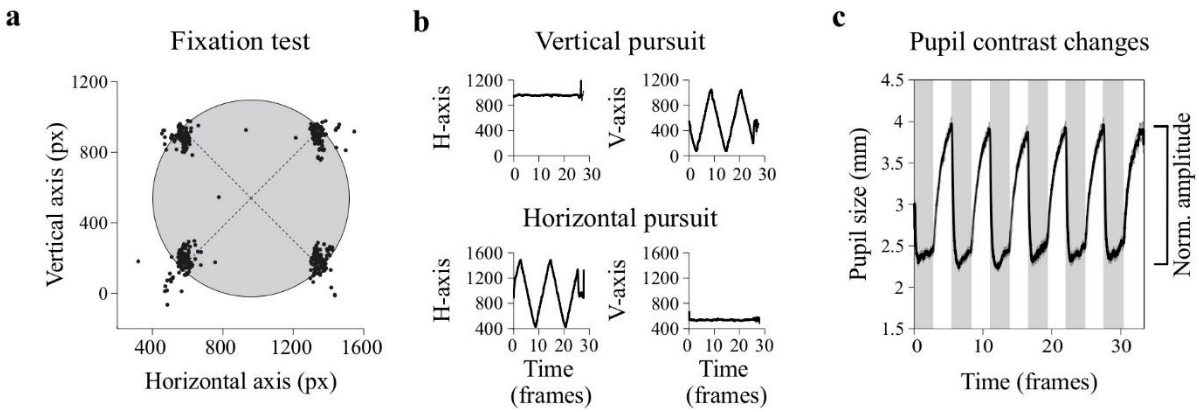

Supplementary Figure S1. Visuomotor task calibration and control procedures.

(a) Four-point fixation test used to calibrate gaze position to screen corners.

(b) Smooth-pursuit calibration in which participants tracked a dot moving horizontally and vertically at 10°/s.

(c) Pupillary light reflex assessment. Average pupil size was measured while the screen alternated between black (0% contrast) and white (100% contrast) during six 35-s cycles, repeated four times under constant ambient illumination (~100 lux). Gray shading indicates white-screen intervals. Data were used to normalize pupil size across participants.

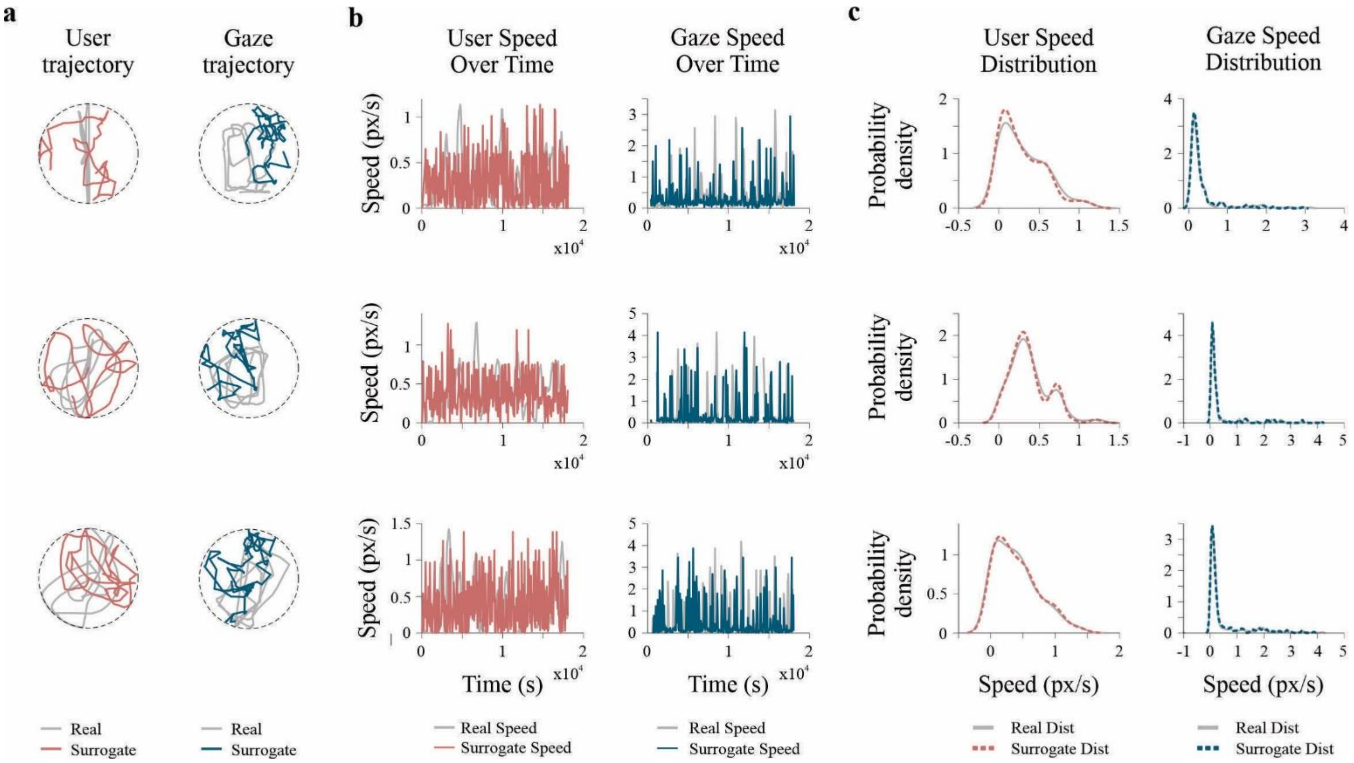

Supplementary Figure S2. Construction and validation of the kinematically matched surrogate null model.

Representative examples from three participants (rows) illustrating surrogate generation for joystick trajectories (left column) and gaze trajectories (right column). To dissociate predictive alignment from passive kinematics, empirical trajectories were compared to a memoryless correlated random walk. The surrogate preserves each participant's instantaneous speed and turn-angle distributions but randomly permutes their temporal order, thereby eliminating systematic coupling to the moving target while maintaining local kinematic structure.

(a) Spatial trajectories. Real trajectories (gray) are shown within the circular arena (dashed boundary). Surrogate trajectories (dusty rose: joystick; deep blue: gaze) start from the same initial position but evolve as temporally shuffled random walks, diverging from target-directed paths while preserving local motion statistics.

(b) Temporal decoupling. Instantaneous speed over time. The surrogate contains the same accelerations and decelerations as the real trace, reassigned to randomized time points, thus preserving kinematic magnitude while removing temporal alignment.

(c) Kinematic preservation. Kernel density estimates of instantaneous speed across the trial show strong overlap between real (gray) and surrogate (colored dashed) distributions, confirming preservation of global speed statistics and variance.

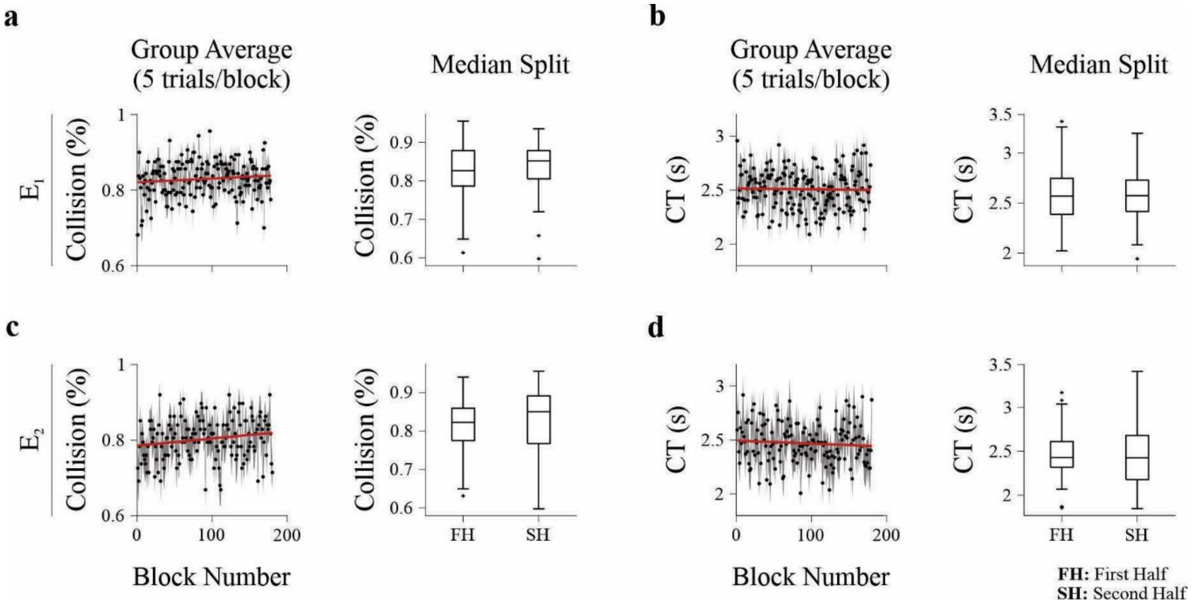

Supplementary Figure S3. Stability of Interception Performance Across the Experimental Session (E_1 and E_2). (a–b) Experiment 1 (E_1). (c–d) Experiment 2 (E_2).

(a) Collision success rate for E_1. Left: Group-averaged percentage of successful interceptions computed in consecutive, non-overlapping blocks of five trials. Shaded areas indicate ±1 SEM across participants. Linear regression on block-averaged data revealed no significant temporal trend across the session (m = 0.0001, R^2 = 0.003, p = 0.43). Right: Median-split comparison of mean success rate between the first and second halves of the session (per participant). A paired t-test showed no significant difference ($t(31)$ = −0.22, p = 0.83).

(b) Collision time for E_1. Left: Block-averaged interception times showed no significant linear change over the session (m = −0.0003, R^2 = 0.006, p = 0.30). Right: Median-split analysis confirmed no significant difference between early and late session interception times ($t(31)$ = 0.89, p = 0.38).

(c) Collision success rate for E_2. Left: Block-averaged success rates showed no significant linear trend (m = 0.0001, R^2 = 0.022, p = 0.06). Right: Median-split comparison revealed no significant difference between the first and second halves of the session ($t(19)$ = −0.47, p = 0.64).

(d) Collision time for E_2. Left: Block-averaged interception times showed no significant change over time (m = −0.0011, R^2 = 0.073, p = 0.26). Right: Median-split analysis confirmed no significant difference between early and late session collision times ($t(19)$ = 2.07, p = 0.152).

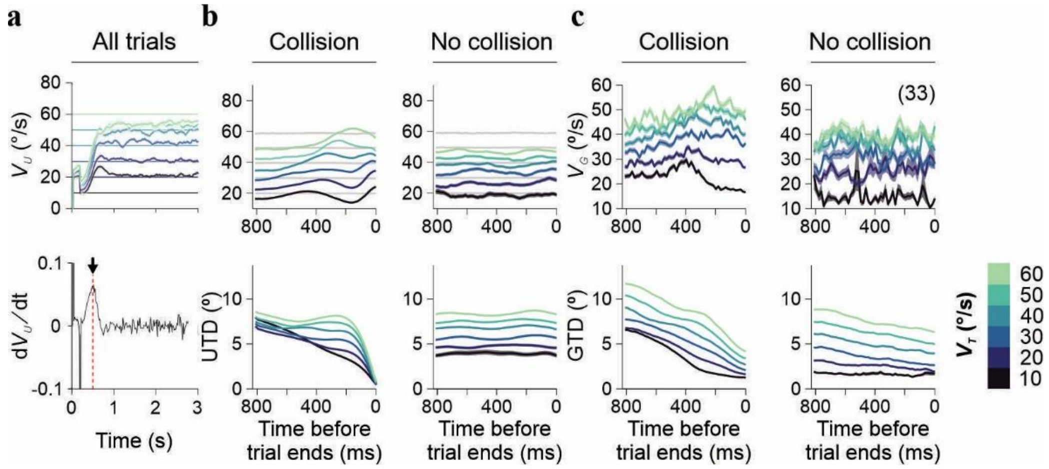

Supplementary Figure S4. Speed adjustment and spatial convergence during interception.

(a) User speed (v_U) as a function of target speed (v_T = 10–60°/s), shown as trial-averaged traces. Lower panel shows the time derivative of v_U, with an early peak (~650 ms) indicating rapid speed alignment.

(b) User speed (upper panels) and user-to-target distance (UTD; lower panels) during the final 800 ms of trials, shown separately for collision and no-collision outcomes.

(c) Gaze speed (v_G; upper panels) and gaze-to-target distance (GTD; lower panels) over the same interval. Successful trials show tighter speed matching and spatial convergence than unsuccessful trials. Data from Group E_1 (n = 33 participants, 900 trials each).

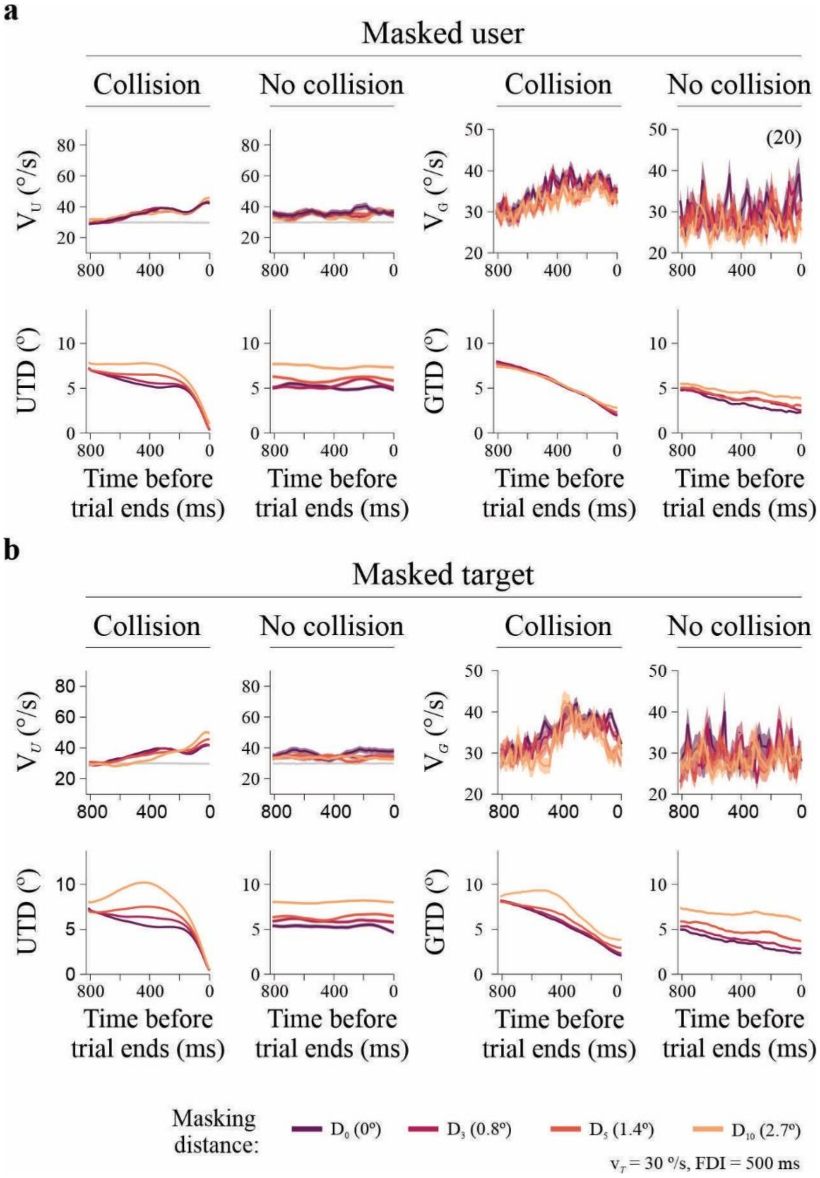

Supplementary Figure S5. Effects of visual occlusion on visuomotor parameters.

(a) Masking the user-controlled cursor at four occlusion distances. Shown are v_U, UTD, v_G, and GTD for collision and no-collision trials. Performance was largely preserved, with minor increases in spatial error.

(b) Masking the target at the same distances. Target occlusion reduced v_U and v_G and increased UTD and GTD, particularly in no-collision trials. Data from Group E_2 (*n* = 20 participants, 800 trials each).

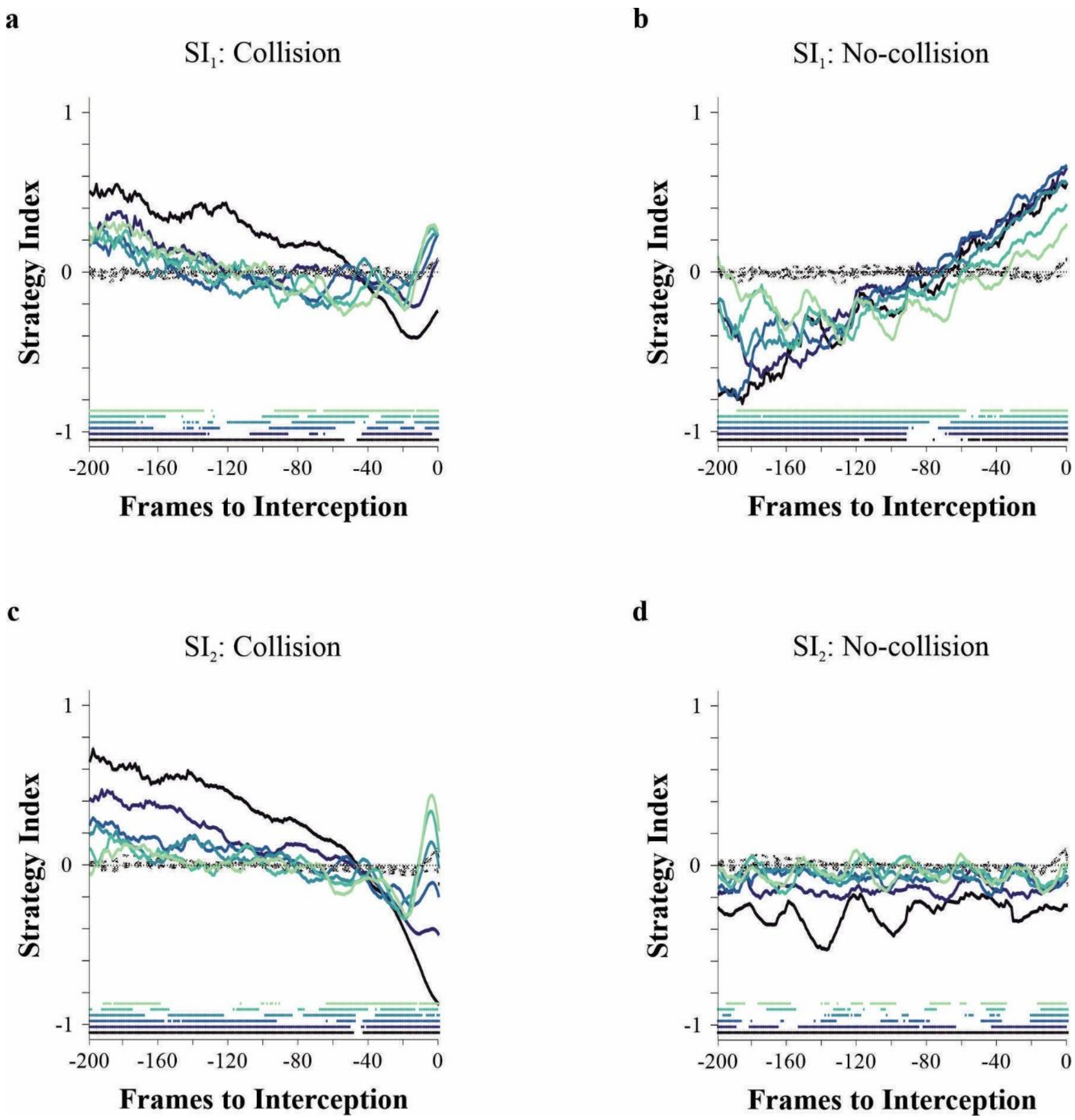

Supplementary Figure S6. Validation of gaze (SI_1) and manual (SI_2) strategy indices against a kinematically matched surrogate null model across target speeds.

Time-resolved comparisons between real strategy indices (solid lines) and the speed-matched surrogate (dashed lines), aligned to trial end (Frame 0). The surrogate was generated using a correlated random walk that preserved each participant's instantaneous speed distribution while temporally decoupling behavior from target motion. Curves are color-coded by target velocity (v_T = 10–60°/s). Colored markers along the lower axis indicate frames at which real and surrogate traces differed significantly (paired t-tests, $p < 0.05$).

(a) SI_1 – Successful (collision) trials. Real gaze indices exceeded the surrogate baseline at multiple target speeds, indicating predictive spatial alignment beyond preserved kinematics. Significant deviations were observed at selected velocities (v_T = 10, 20, and 60°/s; $p < 0.001$).

(b) SI_1 – Unsuccessful (no-collision) trials. Gaze indices showed robust negative deviations from the surrogate across all target speeds (v_T = 10–60°/s; all $p < 0.001$), reflecting systematic spatial lag relative to the target.

(c) SI_2 – Successful (collision) trials. Manual indices were higher than the surrogate across most target speeds (v_T = 10–50°/s; $p < 0.05$), with larger effects at lower velocities (v_T = 10°/s: $t(97) = 17.59$, $p < 0.001$, $d = 1.78$), consistent with active alignment beyond intrinsic kinematics.

(d) SI_2 – Unsuccessful (no-collision) trials. Manual indices exhibited strong negative deviations from the surrogate across all speeds (all $p < 0.001$; effect sizes up to $d = -2.74$), indicating persistent lag rather than random motion.
